# Stroke program for India

**DOI:** 10.4103/0972-2327.61273

**Published:** 2010

**Authors:** Nishant K. Mishra, Satish V. Khadilkar

**Affiliations:** Acute Stroke Unit, Western Infirmary and Faculty of Medicine, Department of Medicine and Therapeutics, University of Glasgow, 44, Church Street, Glasgow, G11 6NT, Scotland; 1 Department of Neurology, Grant Medical College and Sir JJ Group of Hospitals, Byculla, Mumbai 400008, India

**Keywords:** Stroke, epidemic, India, national program, strokology

## Abstract

India is silently witnessing a stroke epidemic. There is an urgent need to develop a national program towards “Fighting Stroke”. This program should be specific to our national needs. In order to recommend on who should lead an Indian fight-stroke program, we examined the published opinions of stroke clinicians and the official documents on stroke care training abroad. We identified the resources that already exist in India and can be utilized to develop a national fight-stroke program. Through a review of published literature, we noted different opinions that exist on who would best manage stroke. We found that because stroke is a cardiovascular disorder of the central nervous system, its management requires a multi-disciplinary approach involving clinicians with background not limited to neurology. India has very few neurologists trained in stroke medicine and they cannot care for all stroke patients of the country. We propose a mechanism that would quickly put in place a stroke care model relevant in Indian context. We recommend for tapping the clinical expertise available from existing pool of non-neurologist physicians who can be trained and certified in stroke medicine (Strokology). We have discussed an approach towards developing a national network for training and research in Strokology hoping that our recommendations would initiate discussion amongst stroke academicians and motivate the national policy makers to quickly develop an “Indian Fight Stroke Program.”

## Introduction

Stroke is a major cause of mortality worldwide and commonly occurs amongst elderly.[1–3] Indian population is relatively young [Indian population≥60 years: 7.5%[4]]compared to the west [e.g. British population aged ≥65 year[5]], but the stroke in India has already attained epidemic proportions [annual incidence of stroke: 13 per 100 000 in 1969-7[6] and 145 per 100 000 per year during 2003-05[7] and 2005-06].[3,8–10] Stroke is a major health issue not only because it is the third major cause of death but also because it leaves patients with several residual disabilities like physical dependence, cognitive decline, dementia, depression, and seizures. The costs involved in caring for these patients are enormous and have adverse social implications. Hence, a quick action to control the stroke epidemic is required so that the now-young Indians are prevented from developing stroke by the time they reach the peak of their life span.[3,11,12]

Stroke mortality rate in India is 22 times that of malaria and 1.4 times of tuberculosis [stroke mortality rate (1990 estimate): 73 per 100 000 persons per year].[13] But, whereas there are national programs meant for eradicating malaria and tuberculosis, there is hardly any national effort to “Fight-Stroke.”[13,14] We need to motivate the health policy makers to develop a national program aiming to contain the rising trends of stroke incidence in India. We should aim for a national framework in which stroke patients are offered highest standard of care.[14]

Stroke care requires active participation of clinicians from different disciplines and not merely neurology. Any national program that we initiate would require leaders well trained with knowledge skills and attitude needed to care for stroke patients. Though doctors can best play this role, a stroke care team would also require well equipped and swift ambulance services, trained paramedical and nursing personnel, swift access to radiology, and rehabilitation facilities.

Here, we discuss a model that would be relevant to Indian needs by studying the published opinions of stroke clinicians regarding who would best lead a stroke team. We identified the resources that already exist in India and can be utilized to develop a national fight-stroke program. We anticipate that our recommendations would initiate a discussion amongst Indian stroke experts and motivate the policy makers to quickly develop an “Indian Fight Stroke Program.”

## Who Should Treat Stroke Patients?

A review of the published literature showed that there are differences in opinion regarding who best treats the stroke patients. Neurologists are given preference over non-neurologists because they are well trained to identify and manage neurological conditions. However, it is also argued that “*A “general” physician, a “general” neurologist, a “general rehabilitation specialist” or a general “physical therapist” are not…truly qualified to care for all of stroke-related aspects*”.[15] Those arguing against the monopoly of neurologists in stroke care note that a specialist neurologist training is not entirely necessary to become a stroke physician. This is because the assessment of stroke disability can simply be undertaken employing tools like NIHSS.[16] Management of patient's fluid and electrolytes or his risk factors [like hypertension and diabetes] is better taken care of by an internist than a neurologist.[17] About 15% of acute stroke presentations are stroke-mimics and even though the neurologist would be better placed to clinically identify them, physicians can also sufficiently be trained to diagnose stroke mimics. Modern neuroimaging such as CT and MRI have further simplified differentiation of non-stroke brain lesions from stroke, skills of which can easily be acquired.[16,17] KR Lees[16] argues that “*the deficit in stroke is neurological but the cause is vascular, complications are medical, and treatment should be multidisciplinary: no single aspect is overriding*”. Even, the stroke stalwart, Louis Caplan emphasizes a need of “*interest, training, and experience in caring for stroke patients*” when commenting in favor of neurologists.[18]

We acknowledge that “secondary prevention is a long-term activity” and very “few neurologists play a direct practical role in the management of blood pressure or atrial fibrillation, use of statins, PFO closure, carotid stenting, or endarterectomy”.[14] Hence, there is a need of “holistic care, expertly delivered” in stroke and “no one speciality over-rides the other in stroke care”.[16]

A guideline from Association of British Neurologist published in 1994 had suggested that stroke patients need not necessarily be seen by a neurologist,[17,19,20] and the recommendation was supported with the argument that “*there are about 200 whole- time equivalent consultant neurologists in the UK where every year about 130 000 people have a first or recurrent stroke. If neurologists are to see all strokes, as Bladin et al. recommend,*[17] *rather than some strokes which we recommend, then every neurologist would have to see about 14 stroke patients every week. Most consultant neurologists in the UK see 15-30 new patients a week and it would be ludicrous if 14 of these were stroke patients when so many other disorders from the common (headache, epilepsy, multiple sclerosis) to the rare (polymyositis, myasthenia gravis, syringomyelia) require neurological skills”*[19,20] This argument was relevant in the context of UK, and it did not imply that patients could not go to neurologists.

Regardless of the speciality the stroke clinician comes from, she/he is expected to have an excellent knowledge of neurology [like neuroanatomy, stroke semiology, its rehabilitation, etc] and also competency in managing the cardiovascular and cardiopulmonary disorders that are often encountered in them. The stroke physicians should also be competent in undertaking a long-term prevention and follow-up of their patients [e.g. managing INR, identification of aetiology in atypical stroke, issues related to end-arterectomy, etc], and must be aware of the newer findings from stroke research in order to be able to offer highest standard of care. We realize that no single clinical speciality can replace the other in the realms of stroke management, and a specific training in stroke care is desirable (even for the neurologists).

### Training in Stroke Care: Global perspective

Regardless of the controversies surrounding the question “who would best manage the stroke patients”, every country has framed its national policy depending on the magnitude of the health problem it faces and the resources that are available to it.[15–22]

In United States, 4-year neurologists’ training is available soon after the medical school. Those interested in stroke undergo a stroke fellowship. These fellowships give emphasis on clinical research along with a structured clinical training program. The training leads to competencies in delivery of stroke care and includes acute care, neuro-imaging, epidemiology, Transcranial Doppler, neurocritical care, etc.[23–27] Further, those interested specifically in researches are eligible to undertake a PhD while still in medical school [called Medical Scientist Training Program leading to MD-PhD]. Several organizations offer possibilities to undertake researches in stroke medicine. Canada has a similar model.

In European Union, the training in stroke care varies across its member states. Most of the countries depend on their neurologists for the stroke care and have incorporated stroke training as an important element of their neurologist training program. In UK, stroke patients are not compulsorily required to be seen by neurologists [19,20] and stroke is developed as a sub-speciality.[28] UK lacks in sufficient number of neurologists[29]  stroke trainees are derived from among general physicians, geriatricians, clinical pharmacologists, cardiologists, etc.[30]

In addition, there are several post-graduate training programs [MSc/MD] run in Europe that is available to any physician interested in stroke. For example, the European Stroke Organisation runs “European Masters′ Programme for Stroke Medicine” and a yearly stroke summer schools that allows participation of stroke physicians from its member states.[15,31]

In addition, in Europe, the medical students have opportunities to exchange programs with other institutions that allow rotation in departments where the faculties are active in specific researches (for example, in stroke). These rotations are given credits and allows students to have exposures to best clinical practices from centres of excellence.

## Recommendations for Indian “Fight-Stroke” Initiative

### Who should manage stroke in India?

In India, stroke is a major health problem and needs urgent attention of its policy makers. There are very few neurologists in India, mostly all are pre-occupied with excessive workload of non-stroke neurological patients.[32] An estimate suggests that 3 million Indians are served by every neurologist in the country.[33] Khadilkar *et al*. estimated that 50% neurologist see 10-30 patients per day [and another 15% neurologists even more than 50 patients every day].[32] Indian neurologists have a very busy clinical practice compared to UK (where the neurologist consultants see 15-30 new patients a week) or USA.[19,34,35] In these dire situations when Indian neurologists are already burdened with excessive neurological workload, it would not be desirable to compel them see only stroke patients. Instead, like in UK, the physicians from other relevant clinical backgrounds should be encouraged to undertake training in stroke and function as stroke specialists in community.[19,20]

### National Stroke Care Model

We believe that Strokology as a speciality is relevant in Indian context and should be developed as a speciality. This would be an important step toward creating a workforce needed to lead the proposed stroke program. We believe that this will have desired impact in controlling the stroke epidemic.

### Referral Centre for Stroke Sciences

The first step toward this initiative would be creation of a National Referral Center for Stroke Sciences. This would be an apex organization with a mandate to undertake clinical researches; develop national guidelines and teaching material on stroke care; conduct examinations and certify the strokologists and other professionals working in stroke care group; undertake international co-operation in stroke; liaise with government bodies and universities, and create opportunities for young stroke physicians to conduct researches relevant in Indian context. Adequately regulated academic-industry partnership may be promoted for stroke research, as specific Indian data on stroke care would result in clinical evidence relevant to stroke management of Indian population. Participation of specifically trained strokologists in this initiative would be in national interest and would be promoted.

### Role of Indian Stroke Leaders in National Program

There are several excellent stroke neurologists in India who are in active clinical practice and academic roles with affiliations in public and private hospitals. They have had post-doctoral training in stroke mostly abroad and are currently members of several learned neurology societies like Indian Stroke Association [www.stroke-india.org]. We believe that by seeking their co-operation, policy makers can design a comprehensive program to contain the stroke epidemic.

### Administration of Stroke Training to Clinicians

Our stroke academicians in co-operation with international colleagues and organizations could work together to develop a curriculum needed to train physicians. The physicians would belong to disciplines as deemed suitable by policy makers. We believe that any medical graduate with atleast 2 years of training/work experience in general medicine should be eligible to undergo a stroke training and certification. Those currently in training would undergo additional rotations whilst still pursuing their existing training program [MD or DNB programs] and obtain an add-on certification in stroke. The Universities can introduce master's level postgraduate courses and affiliate hospitals that have necessary infrastructure. Or, a rotation in a stroke unit of pre-specified duration should lead to an add-on certification for the neurologists. We believe that the national referral center for stroke care should centrally monitor the training and assist in recognizing the areas that each trainee must be trained in. The Universities offering stroke care training may want to take some administrative responsibilities and probably in association with the affiliated stroke team may function as regional referral centre for stroke care.

## Strokology

*A Speciality*. A “Strokologist” is expected to have sufficient knowledge skills and attitude needed for the prevention management and rehabilitation o f stroke patients.[36] Strokologist would acquire these by rotating through stroke units and other relevant specialities (e.g. neurology, rehabilitation, cardio-vascular risk factors management clinics, neuro-radiology etc). We suggest that any stroke training should also require accomplishment of an original research project, as we need to gradually develop a stroke care program specific to Indian requirements. The manner in which the training is offered to each physician should be guided by the identification of clinical areas that the physician has not had training in his parent speciality.[30] Length of training should depend on his previous qualification. We believe that the program should be tailor made based on pre-specified guidelines while taking into consideration his/her background speciality. When trained and certified, the physician would normally practise his background speciality, and he would also be capable of confidently handling acute stroke based on national stroke guidelines.

We also expect that Stroke-trainee would devote certain duration of his training by participating in community awareness and mitigation of the superstitions and/or hold regular seminars for the physicians and other health care workers on current stroke guidelines or conduct the Indian/ World Stroke Day programs.[14]

## Catch-Them-Young Policy

Usually strokologists would be the physicians who already hold a postgraduate qualification. We would however encourage a “catch-them-young” policy offering excellent mentoring of the young medical graduates who show potentials as policy maker or a clinical scientist in stroke. For these candidates, we recommend create opportunities to undertake a longer comprehensive clinical training accompanied with research culminating in a clinical doctorate qualification.

**Incorporation of Stroke Medicine in Undergraduate Medical Education Curriculum:** Young medical students should compulsorily be evaluated for their knowledge of stroke care, its prevention, and rehabilitation, and therefore the relevant topics on stroke should be incorporated in their curriculum. [36] Fear of neurology as a difficult subject should be removed from young minds. Opportunities have to be created for them in order to undertake short-term clinical research projects in a stroke center through a national funding scheme. Just like every medical student knows how to care for malaria, leprosy, and tuberculosis owing to relevant national programs, they should also be aware of National “Fight-Stroke” program and means thereof.[36]

**Preventive Medicine:** Stroke occurs in a relatively younger population amongst Indians. This is probably because the cardiometabolic risk factors emerge at a younger age. [37–41] Whereas there are certain risk factors that are rare but known to occur amongst Indians [like squatting resulting into hypertension.[42,43] the common risk factors (e.g. Diabetes, hypertension, metabolic syndrome, smoking, obesity, etc) remain similar to those described elsewhere in the world.[44–47] These risk factors are a major burden on Indian society. These are so common now that India is described as a world capital of diabetes and hypertension. [48,49] Current estimates suggest that every fifth diabetic or a hypertensive in the world is an Indian.[48,49] Insulin resistance and related metabolic syndromes are emerging as major health problems in India resulting into cardiovascular diseases[50–53]. Hence, there is an urgent need to control cardiometabolic risk factors in our population. We recommend that cardiovascular risk factor clinics should be run in conjunction with stroke care and relevant specialities should be integrated. Stroke physician must be trained in prevention and management of all these risk factors.

## Anticipated Impact of “Strokology” as a Speciality in India

The discipline would result in a new generation of well-trained strokologists who would function as leaders in stroke care, research, and policy making. They would impart a standard care to stroke patients based on the national guidelines emanating from apex referral center. They would reduce the burden of stroke care on neurologists. Owing to specific training in stroke care, we expect them to be better trained to care for these patients. Academically inclined strokologists would be caught young to advance science through research and generate data specific to Indian context. This is important, as the practice of stroke care in India is based on evidences generated from the non-Asian population. By compulsorily incorporating elements of community awareness and conduct of seminars, they would spread the message that Stroke is not a “stroke of god” and further that “in stroke, there is hope”.[14] Because they would also be practicing their primary speciality, they would come across patients who have not yet had stroke but harbor risks factors that may lead to it. In these cases, these strokologists would be better placed to undertake preventive measures and educate their patients to lead healthy lifestyle. They would be able to pursue long term follow-up of these patients and would reduce the risks of other clinical events related to stroke e.g. stroke recurrence, uncontrolled INR, etc.

Further, neurologists are mostly based in cities. They have to care not only for stroke patients but also for those suffering from other neurological disorders. We believe that by incorporating the physicians in the national umbrella of Strokology, we would be tapping their potential in a national fight-stroke program and be quickly able to create much-needed work force to participate in it. Because the physicians practice closer to the community, stroke care would reach closer to the general population. Because of the widespread permeation of information technology, stroke physicians would easily be able to contact the stroke neurologists at referral centres in cases where the decision making is difficult.

## Stroke Training for Nursing, Paramedical Staff and Others

Stroke care is a combined effort of all stroke team members, and therefore their training and certification is also needed. Their training would vary depending on the roles they would play in the team. A mechanism to incorporate training of these personnel in stroke care program should be evolved.

## Conclusion

An Indian “Fight-Stroke” Program specific to our needs is urgently needed. Stroke is a cardiovascular disorder of the central nervous system and its management requires skills that overlap with several clinical specialities. Though it is generally believed that the neurologists are well trained to treat stroke patients even they require training in vascular neurology to develop an attitude specifically needed in stroke care. Stroke management often requires participation of clinicians belonging to other specialities e.g. cardiology, clinical pharmacology, geriatrics, critical care, endocrinology, etc, and therefore we are of the opinion that Strokology as a new speciality deriving its trainees from relevant specialities would be useful to create a huge workforce needed to run the Indian Fight Stroke Program. Training of other members of stroke team can also be undertaken by encouraging respective societies to co-ordinate their training program with the proposed stroke initiative.
